# Early Experience with Synthetic Monofilament Polydioxanone Mesh in Primary Breast Reconstruction: A Propensity Matched Analysis

**DOI:** 10.1055/a-2850-0163

**Published:** 2026-06-17

**Authors:** Puja Jagasia, Reena Sulkar, Anna Whitney, John Y.S. Kim

**Affiliations:** Division of Plastic Surgery, Northwestern Memorial Hospital, Chicago, Illinois, United States

**Keywords:** breast reconstruction, synthetic mesh, implant-based breast reconstruction, breast implants, acellular dermal matrix

## Abstract

**Background:**

Polydioxanone (PDS) mesh is an emerging technology for prosthetic breast reconstruction. However, there is a paucity of data on its safety and efficacy in primary breast reconstruction.

**Methods:**

Demographic and outcomes measures from consecutive, immediate prosthetic breast reconstructions using PDS or acellular dermal matrix (ADM) from a single surgeon were evaluated on a *per-breast* basis between 2013 and 2023. Propensity matching based on demographic variables was conducted. Binary logistic regression analyses were performed to assess the impact of the mesh.

**Results:**

A total of 796 breast reconstructions were identified, and propensity matching yielded 192 ADM and 48 PDS mesh reconstructions. There was no significant difference with respect to age, American Society of Anesthesiologists (ASA) status, smoking history, diabetes, mastectomy indication, radiation, chemotherapy, expander plane, and mastectomy type between propensity matched cohorts. Body mass index (BMI) varied by 2.1 points (ADM: 25.8 vs. PDS: 23.7, *p* = 0.01). Mean follow-up time was 13.5 ± 2.7 months. The propensity-matched PDS mesh cohort had lower rates of any complication (ADM: 23.9% vs. PDS mesh: 4.2%, *p* = 0.003), seroma (ADM: 11.4% vs. PDS mesh: 0%, *p* = 0.01), expander exposure (ADM: 12.5% vs. PDS mesh: 0%, *p* = 0.01), complication-associated expander removal (ADM: 9.9% vs. PDS mesh: 0%, *p* = 0.02), and mastectomy flap necrosis (ADM: 14.0% vs. PDS mesh: 4.2%, *p* = 0.05). PDS mesh, compared to ADM, was independently associated with decreased risk of any complication (OR: 0.443, *p* = 0.02), seroma (odds ratio [OR]: 0.456, *p* = 0.03), expander exposure (OR: 0.277, *p* = 0.03), complication-associated expander removal (OR: 0.211, *p* = 0.02), and mastectomy flap necrosis (OR: 0.12, *p* = 0.04).

**Conclusion:**

PDS mesh is an alternative to ADM in primary prosthetic breast reconstruction. Rates of complication—including infection, seroma, and explantation—suggest an acceptable safety profile relative to ADM.

## Introduction

Implant-based breast reconstruction (IBBR) is currently the dominant reconstructive approach after mastectomy and comprises over 80% of breast reconstruction procedures. 1 2 Acellular dermal matrix (ADM) is a biological mesh that is used off-label as a support scaffold in over 50% of IBBRs in the United States. 3 4 5 6 Although the use of biologic scaffolds such as ADMs has been associated with favorable aesthetic outcomes and provides additional tissue support in the setting of direct-to-implant and pre-pectoral two-stage IBBR, they have also been associated with complications such as seroma and infection. 7 8 9 10 11 12 13 14

Recently, synthetic absorbable meshes have been gaining traction as an alternative to biologic meshes. 5 9 13 Their value proposition lies in the potential for (1) less foreign body reaction (with concomitant quicker absorption and possible decreased inflammatory sequelae of seroma and infection), (2) cost savings, and (3) consistent product specifications not subject to varying circumstances of donor source (thickness, age, and anatomic location). 14

Two-stage IBBR involves placement of a tissue expander (TE) followed by an exchange surgery to place the final breast implant. Use of a scaffolding material at the time of TE placement can be beneficial, especially for pre-pectoral TE placement, which does not have the added support of the pectoralis muscle. Synthetic mesh options currently on the market vary in regard to their handling, chemical attributes, and time to resorption. 14 Ideally, a resorbable scaffold would persist long enough to support the formation of a durable tissue pocket, but eventually incorporate into the local tissue in a timely manner to prevent long-lasting foreign body response (FBR) and associated complications.

A recent review of the Mastectomy Reconstruction Outcomes Consortium (MROC) registry demonstrated a mean time of 5.5 months between TE placement and exchange to implant. 15 Monofilament polydioxanone (PDS) mesh (DuraSorb®, Integra LifeSciences, Princeton, NJ, United States), based on the commonly used PDS suture, has demonstrated an absorption profile consistent with this timeline, with full resorption between 6 and 12 months. 16 As it resorbs, a thin, pliable layer of capsule is integrated into the soft tissue. 16 Previous studies show that PDS mesh is an adjunct that can be safely and effectively used in multiple types of breast surgeries. 6 17 18 19 20 However, to the authors' knowledge, no studies have directly compared the use of PDS mesh with ADM for the first stage of two-stage IBBR.

Accordingly, the authors endeavored to evaluate outcomes of PDS mesh in primary breast reconstruction versus ADM with a single surgeon's experience over a decade, controlled with propensity matched cohorts. We hypothesize that PDS mesh is a safe alternative to ADM in primary breast reconstruction with similar complication rates.

## Methods

### Data Collection

After Institutional Review Board approval (STU00213421), a prospectively maintained database of all IBBRs performed by J.K. was queried for consecutive patients undergoing immediate primary expander-based breast reconstruction with any type of mesh scaffolding in a 10-year span between January 2013 and December 2023. Prior to March 2021, all prosthetic reconstructions were performed with ADM, and after December 2021, all prosthetic reconstructions were performed with PDS mesh. The intervening period was a mixed cohort of ADM and PDS and was excluded to minimize the risk for bias in surgeon decision-making. The database was maintained, and all analyses were performed on a *per-breast* basis. Demographics of interest included age, body mass index (BMI), American Society of Anesthesiologists (ASA) classification (I, II, or III), radiotherapy, chemotherapy, diabetes, smoking status, plane of expander placement (pre-pectoral or subpectoral), type of mastectomy (nipple-sparing mastectomy [NSM] or skin-sparing mastectomy [SSM]), and indication for mastectomy (prophylactic or therapeutic). Complications of interest included prosthetic migration, prosthetic rupture, infection, seroma, hematoma, exposure, complication-associated expander removal, and mastectomy flap necrosis requiring operative intervention. Infection was defined as cellulitis requiring antibiotics or surgery. Seromas were defined as clinically evident fluid collections requiring percutaneous or open drainage. Flap necrosis was defined as skin necrosis requiring sharp debridement. Wound dehiscence was defined as the separation of the mastectomy incision requiring dressing changes or operative intervention. Explantation was defined as the all-cause removal of expanders or implants. These complications were assessed during the first stage of IBBR.

### Surgical Technique

**Supplementary Video S1** Video demonstrating the placement of mesh in subpectoral reconstruction.


**Supplementary Video S2** Video demonstrating the wrap technique **employed** by the senior author for pre-pectoral reconstruction in cases where nipple-sparing mastectomies were performed.


**Fig. 1:**
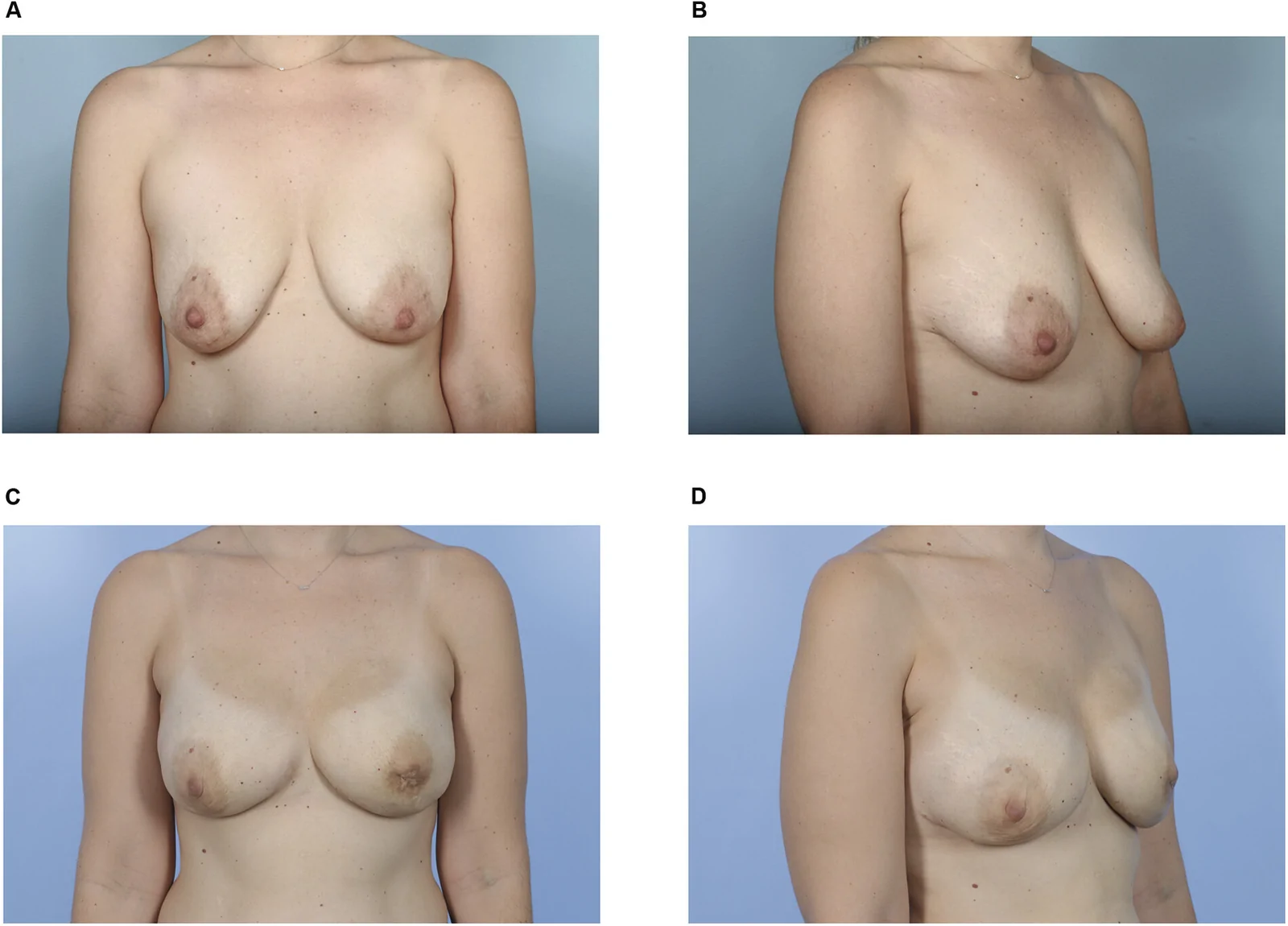
( **A, B** ) PDS mesh showing good shape concordance with the underlying prosthesis in a pre-pectoral reconstruction with ( **A** ) the mastectomy flap reflected, and ( **B** ) the mastectomy flap covered over mesh. PDS, polydioxanone.

**Fig. 2:**
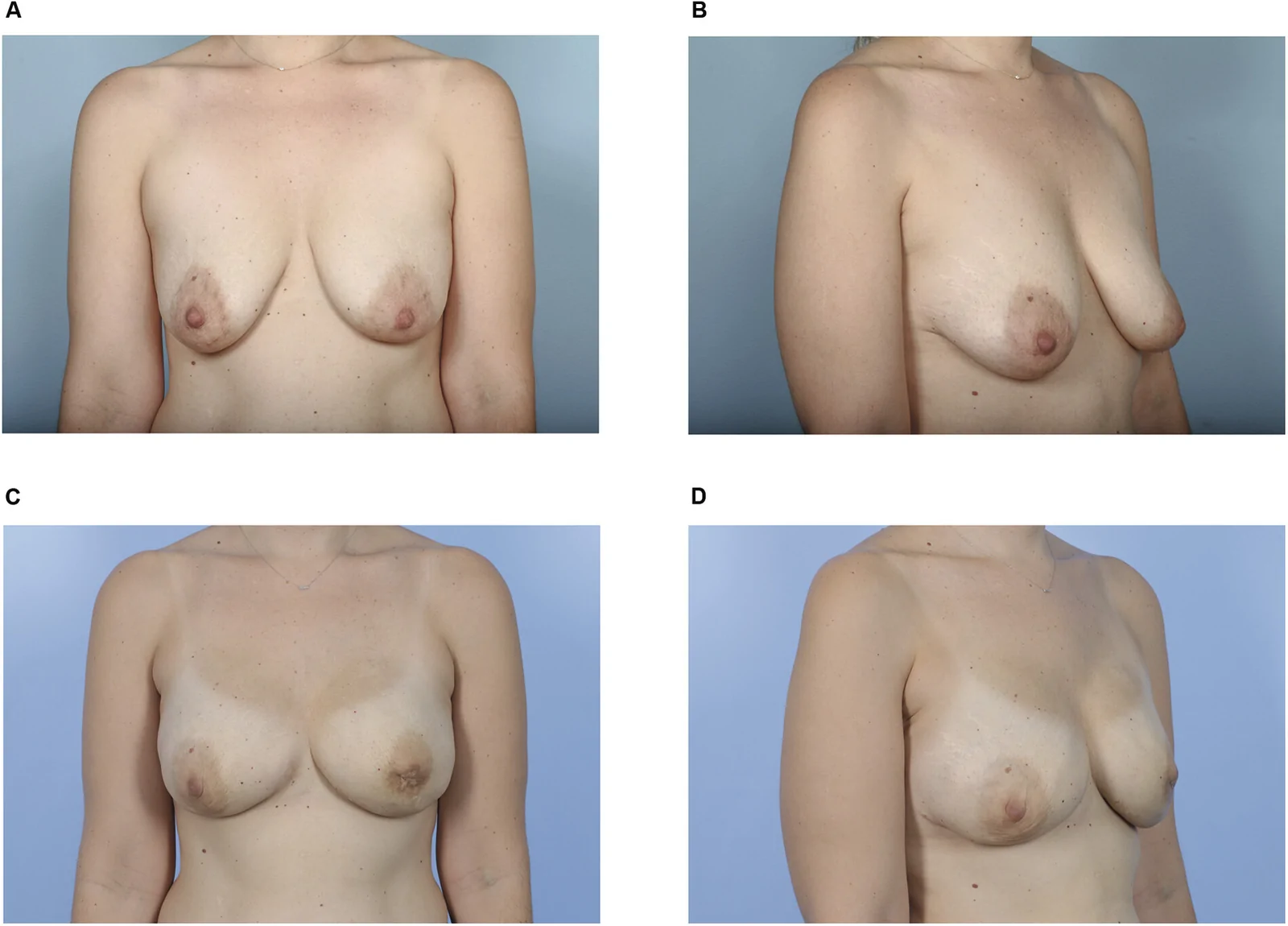
( **A–D** ) Preoperative ( **A, B** ) and 9 months postoperative ( **C, D** ) images from a 34-year-old woman who underwent two-stage expander to implant pre-pectoral breast reconstruction with ADM and 650-mL smooth full profile silicone gel implants after exchange. ADM, acellular dermal matrix.

**Fig. 3:**
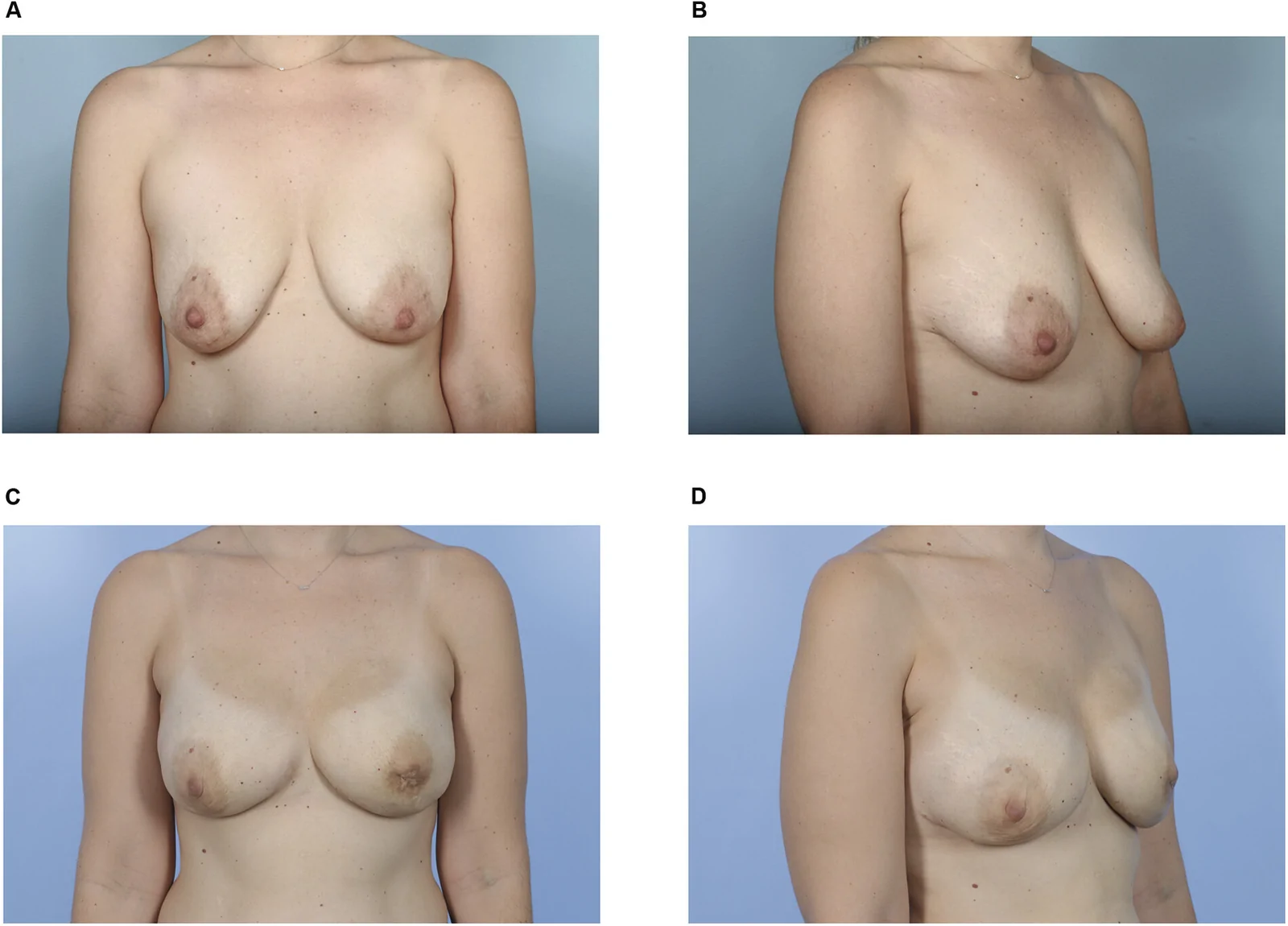
( **A–D** ) Preoperative ( **A, B** ) and 8 months postoperative ( **C, D** ) images from a 30-year-old woman who underwent two-stage expander to implant submuscular breast reconstruction with ADM and 375-mL moderate projection textured, shaped silicone gel implants after exchange. ADM, acellular dermal matrix.

**Fig. 4:**
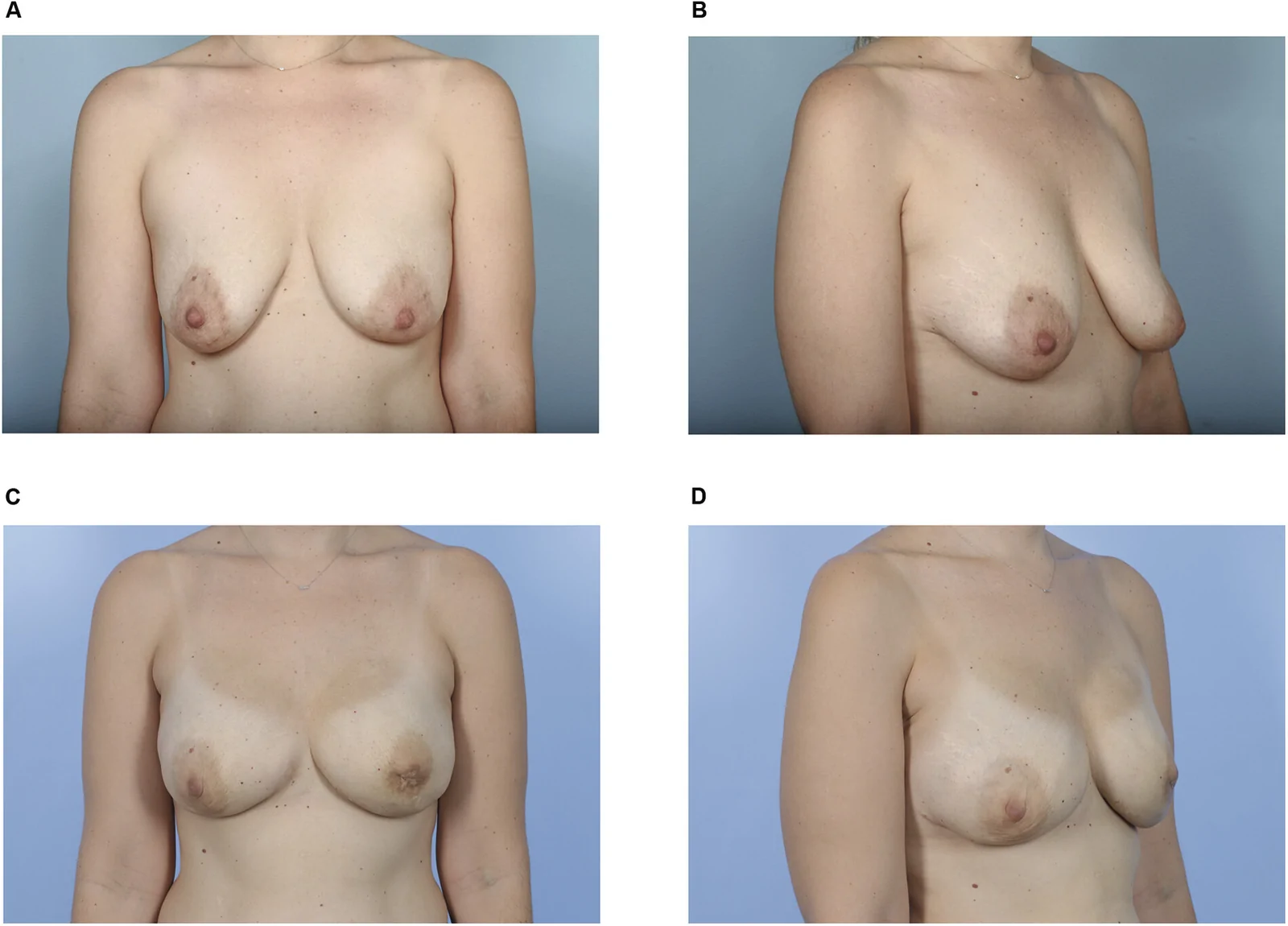
( **A–D** ) Preoperative ( **A, B** ) and 1-year postoperative ( **C, D** ) images from a 48-year-old woman who underwent two-stage expander to implant submuscular breast reconstruction with Durasorb and 450- to 480-mL saline-filled implants filled to 500 mL at the exchange.

**Fig. 5:**
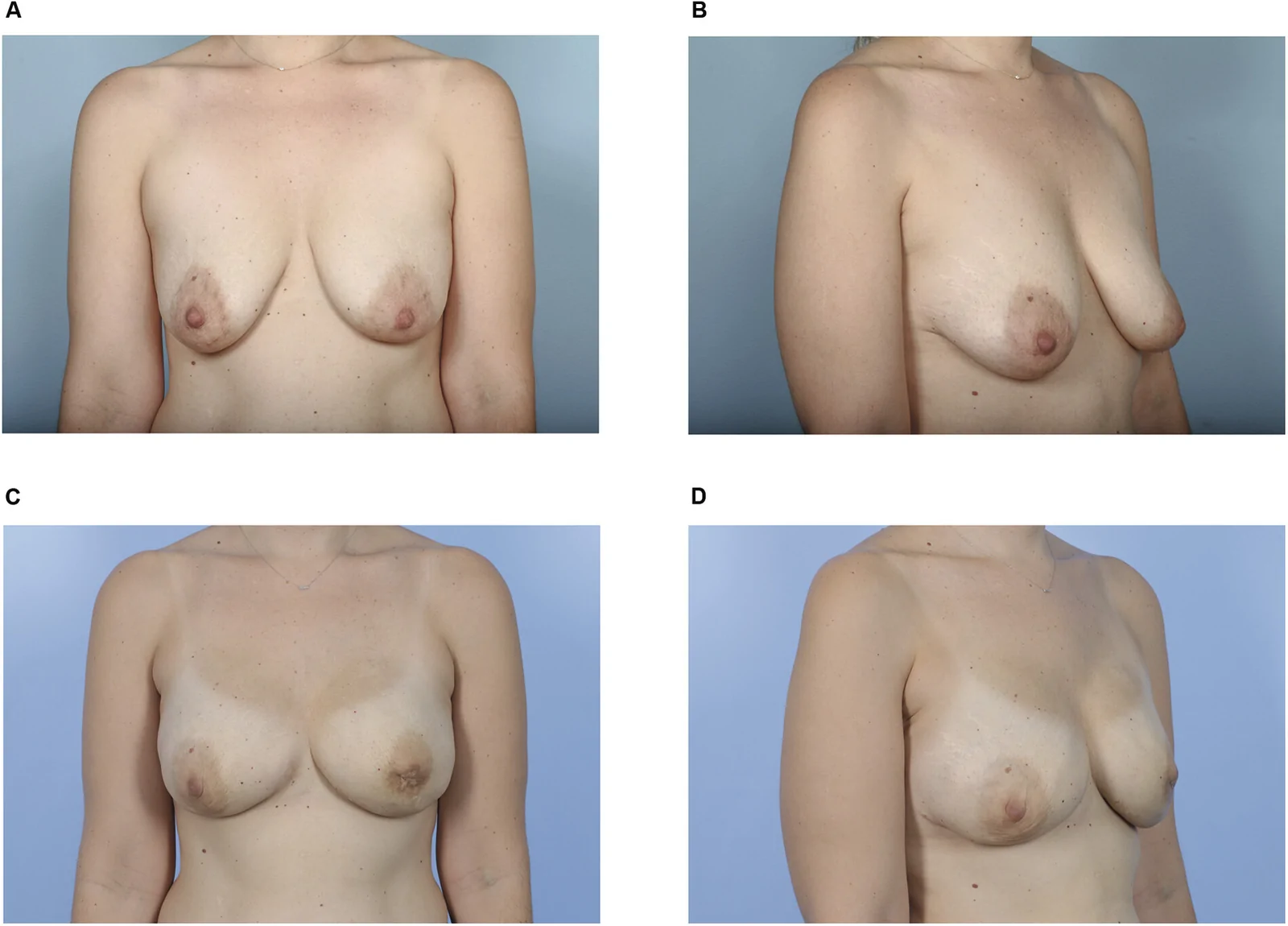
( **A–D** ) Preoperative ( **A, B** ) and 1-year postoperative ( **C, D** ) images from a 39-year-old woman who underwent two-stage expander to implant submuscular breast reconstruction with Durasorb and 540 mL smooth low plus profile silicone gel implants after exchange.

Subpectoral reconstructions were performed with a standardized technique in which the lower border of the pectoralis was incised and ADM, Alloderm® (LifeCell Corporation, Branchburg, NJ) and FlexHD® (Musculoskeletal Transplant Foundation, Edison, NJ), or PDS mesh was then laid in the ensuing defect and secured with 2.0 suture ( Supplementary Video S1 [available in the online version only]). For pre-pectoral reconstructions, if the mastectomy was skin sparing, an onlay of ADM or mesh was used over the expander (contoured to the size of the expander; Fig. 1A ). Figure 1B demonstrates PDS mesh pliability and optimal concordance with the underlying prosthesis to achieve natural lower pole aesthetics. If a nipple-sparing approach was used, the author's preferred approach is a wrap of ADM or mesh, which is then inserted through an Inframammary fold incision and secured again with 2.0 PDS suture ( Supplementary Video S2 [available in the online version only]). There was no difference in the surface area or cut pattern used between ADM and mesh. Figures 2 and 3 demonstrate preoperative and postoperative images from breast reconstructions with ADM, while Figs. 4 and 5 show preoperative and postoperative images from breast reconstructions with PDS mesh. Informed consent was obtained to use patient photos and information in corresponding figures. At initial placement, TEs were filled intraoperatively with air between 33% and 50% of total expander volume. This did not change throughout the study period.

### Statistical Analysis

Demographics and complication rates were compared between the two cohorts using an independent samples *t* -test for continuous variables and either a chi-square test or Fisher's exact test for categorical variables, as appropriate, based on frequency. The Shapiro–Wilk test was used to assess the distribution of data. Normally distributed data were presented with the mean ± standard deviation, while non-normally distributed data were presented with the median and interquartile range. To decrease selection bias between the two groups, propensity score matching was used. Since the size of the cohorts varied significantly, with the ADM group having over 15 times the number of patients, 1:4 nearest neighbor propensity score matching without replacement was conducted with a caliper width of 0.1. Propensity scores were calculated using logistic regression with covariates of age, BMI, smoking history, ASA status, diabetes, expander plane (pre-pectoral vs. subpectoral), and radiation. Standardized mean differences (SMDs) were calculated to assess the balance between the ADM and PDS mesh cohorts, with the aim of an SMD less than 0.1 for each covariate.

After propensity matching, binary logistic regression analyses were used to account for demographic differences that remained imbalanced and to identify variables that were independently associated with complications. To ensure appropriate use of binary logistic regression analysis, it was confirmed that there were no perfect multicollinear relationships among the independent variables assessed. All continuous variables utilized in the binary logistic regression were tested to ensure a linear relationship with dependent variables, thus satisfying the fundamental assumptions of the model. Hosmer–Lemeshow and c-statistics were calculated to assess the regression model's goodness of fit and discrimination, respectively. For each independent variable, the odds ratio (OR), 95% confidence interval (CI), and *p* -value were reported. For categorical variables with multiple distinctions, such as ASA classification, ASA class I was used as a reference value to calculate subsequent ORs. All statistical analyses were conducted with SPSS version 29.0 (IBM Corp., Armonk, NY), with a *p* -value of less than 0.05 indicating significance.

## Results

The database search yielded information on 796 reconstructed breasts over 10 years. ADM was used in 748 cases, while PDS mesh was used in 48 cases. A demographic summary of the two cohorts on a *per breast* basis can be seen in Supplementary Table S1 (available in the online version only). After propensity matching, 192 patients with ADM and 48 patients with PDS mesh were selected ( Table 1 ). The only demographic factor that varied significantly was BMI, which was an average of 25.8 for the ADM cohort and 23.7 for the PDS.

**Table 1:** Demographic and comorbidity comparison of acellular dermal matrix and polydioxanone mesh propensity matched cohorts

		ADM ( *n* = 192)	PDS mesh ( *n* = 48)	*p* -Value
Age		44.9 ± 10.1	43.0 ± 8.7	0.17
BMI		25.6 ± 5.1	23.7 ± 4.4	**0.02**
ASA status	I	15 (7.8%)	2 (4.1%)	0.14
	II	141 (73.4%)	39 (84.7%)	
	III	38 (19.7%)	7 (15.2%)	
Smoking	Never	144 (75.0%)	34 (73.9%)	0.63
	Current/Former	48 (25.0%)	14 (30.4%)	
Diabetes		8 (4.2%)	0 (0%)	0.22
Mastectomy Reason	Prophylactic	91 (47.3%)	26 (54.1%)	0.39
	Therapeutic	101 (52.7%)	22 (47.8%)	
Radiation		12 (6.3%)	2 (4.2%)	0.26
Chemotherapy		72 (37.5%)	14 (30.4%)	0.22
Expander plane	Pre-pectoral	6 (3.1%)	0 (0%)	0.17
	Subpectoral	186 (96.9%)	48 (100%)	
Mastectomy type	SSM	91 (47.3%)	17 (44.7%)	0.32
	NSM	101 (52.7%)	31 (64.6%)	

Abbreviations: ADM, acellular dermal matrix; ASA, American Society of Anesthesiologists; BMI, body mass index; CI, confidence interval; NSM, nipple-sparing mastectomy; PDS, polydioxanone; ref., reference; SSM, skin-sparing mastectomy.

Next, a comparison of complication rates between the two propensity matched cohorts was completed ( Table 2 ). This showed that the PDS mesh cohort had significantly lower rates of any complication, seroma, expander exposure, complication-associated expander removal, and mastectomy flap necrosis.

**Table 2:** Comparison of complication rates between acellular dermal matrix and polydioxanone mesh propensity matched cohorts

	ADM ( *n* = 192)	PDS mesh ( *n* = 48)	*p* -Value
Mastectomy flap necrosis	27 (14.0%)	2 (4.2%)	**0.05**
Expander migration	1 (0.5%)	0 (0%)	1.00
Expander rupture	2 (1.0%)	0 (0%)	0.78
Seroma	21 (10.9%)	0 (0%)	**0.02**
Infection	11 (5.7%)	0 (0%)	0.07
Hematoma	2 (1.0%)	0 (0%)	0.81
Exposure/Dehiscence	21 (10.9%)	0 (0%)	**0.02**
Capsular contracture	0 (0.0%)	0 (0%)	1.00
Any complication	44 (22.9%)	2 (4.2%)	**0.01**
Complication-associated expander removal	19 (9.9%)	0 (0%)	**0.02**
Elective expander removal	8 (4.2%)	2 (4.2%)	0.89

Abbreviations: ADM, acellular dermal matrix; PDS, polydioxanone.

To determine if these complication rates were lower in the PDS mesh cohort due to differences in demographics and comorbidities rather than scaffolding material, binary logistic regression analyses were performed.

Since the rates of any complication occurring differed significantly between the PDS mesh and ADM cohorts, a binary logistic regression was performed to assess independent predictors of any complication. The model found BMI (OR: 1.166, CI [1.013–1.645], *p* = 0.02) and current or former smoking status compared to no smoking history (OR: 2.319, CI [1.892–3.247], *p* = 0.01) to be independently associated with increased risk of experiencing any complication ( Supplementary Table S2 [available in the online version only]). Factors independently associated with decreased risk of any complication were found to be the use of PDS mesh compared to ADM (OR 0.523, CI [0.093–0.824], *p* = 0.01) and subpectoral expansion plane compared to pre-pectoral plane (OR: 0.281, CI [0.114–0.827], *p* = 0.04).

A binary logistic regression analysis for seroma formation was performed. Factors independently associated with decreased risk of seroma were found to be the use of PDS mesh compared to ADM (OR: 0.414, CI [0.182–0.781], *p* = 0.03) and subpectoral expansion plane compared to pre-pectoral plane (OR: 0.281, CI [0.180–0.557], *p* = 0.01; Supplementary Table S3 [available in the online version only]).

Next, a binary logistic regression analysis to predict expander exposure was completed. As seen in Supplementary Table S4 (available in the online version only), the only independently associated risk factor for expander exposure was BMI (OR: 1.433, CI [1.094–1.883], *p* = 0.01). The use of PDS mesh compared to ADM was independently associated with decreased risk of expander exposure (OR: 0.256, CI [0.193–0.959], *p* = 0.03).

A binary logistic regression analysis of factors associated with expander removal secondary to complications was also completed. The factors associated with increased risk of complication-associated expander removal, shown in Supplementary Table S5 (available in the online version only), were BMI (OR: 1.246, CI [1.134–1.952], *p* = 0.02) and current or former smoking status compared to no history of smoking (OR: 2.103, CI [1.125–4.421], *p* = 0.02). The only factor independently associated with decreased risk of complication-associated expander removal was found to be the use of PDS mesh compared to ADM (OR: 0.201, CI [0.032–0.792], *p* = 0.02).

Factors that were independently associated with increased risk of mastectomy flap necrosis were BMI (OR: 1.194, CI [1.132–1.642], *p* = 0.01), current or former smoking status compared to no history of smoking (OR: 2.827, CI [1.759–4.781], *p* < 0.001), and mastectomy type of NSM compared to SSM (OR: 2.237, CI [1.181–4.014], *p* = 0.01; Supplementary Table S6 [available in the online version only]). The only factor found to be independently associated with decreased risk of mastectomy flap necrosis was use of PDS mesh compared to ADM use (OR: 0.109, CI [0.014–0.901], *p* = 0.03).

Infection rates did not vary significantly between the two cohorts. A binary logistic regression analysis to predict infection found BMI to be an independently associated risk factor for infection (OR: 1.194, CI [1.074–1.267], *p* = 0.01), while subpectoral placement was found to be independently associated with decreased risk of infection compared to pre-pectoral placement (OR: 0.318, CI [0.092–0.832], *p* = 0.03; Supplementary Table S7 [available in the online version only]). Again, it is important to emphasize that the difference in infection rates did not reach statistical significance.

## Discussion

Prosthetic breast reconstruction has continued to evolve over the last decade, with novel techniques and technologies working towards the common goal of enhanced patient outcomes. ADM has helped to improve cosmetic outcomes by allowing more precise control over shape and volume. However, clinical exploration with synthetic meshes as a substitute for ADM continues. This analysis of a single surgeon's experience compared the performance and safety profiles of a PDS monofilament mesh to those of biologic ADM in the first stage of a two-stage IBBR performed immediately after mastectomy. This study analyzed 870 reconstructed breasts over a 10-year span, with 10.1% (88/870) of reconstructions using PDS mesh. Initial analysis revealed that the first stage IBBR reconstruction using PDS mesh has lower rates of mastectomy flap necrosis, seroma, expander exposure, any complication, and complication-associated expander removal compared to reconstruction with ADM. Binary logistic regression analysis showed that the use of PDS mesh compared to ADM was independently associated with decreased risk of all aforementioned complications that varied significantly between the two groups.

The rates of complications in both PDS mesh and ADM cohorts were similar to rates reported in existing literature. 21 22 23 24 While complication rates differ depending on factors such as expander plane, scaffolding material, mastectomy type, and exposure to radiation, studies report overall complication rates between 11% and 46.2%, infection rates between 10% and 28.9%, seroma rates between 6.7% and 15.4%, expander explantation rates between 4% and 19.2%, mastectomy flap necrosis between 5.3% and 15.4%, and expander removal between 8% and 25%. 21 22 23 24

Our finding that the use of PDS mesh was independently associated with decreased risk of seroma formation compared to ADM may, in part, be explained by PDS mesh's decreased FBR. Seroma, or the abnormal accumulation of serous fluid in a dead space, has a well-established pathway to infection and risk of subsequent explantation. 7 25 Jordan et al identified the FBR as one of the etiologies of seroma formation, with increased inflammation during the FBR associated with increased risk of seroma, infection, and explantation. 7 ADMs were initially implemented as a structural support that would revascularize and integrate into the surrounding hypovascular mastectomy milieu. 26 27 However, ADM use—relative to pure submuscular reconstruction—demonstrated increased rates of seroma and infection. 9 28 29 Compared to the FBR to ADM, which peaks 2 to 6 weeks after implantation, PDS mesh is initially relatively inert and does not produce its peak inflammatory response until 12 weeks, at which time it has already absorbed into surrounding tissue. 30 31 32 Additionally, PDS mesh offers the advantage of complete absorption and termination of the FBR by 6 to 12 months compared to ADM, which, rather than fully absorbing, integrates into the local environment and may lead to a sustained FBR that may persist for even years after implantation. 16 27 33 34 Here, the decreased rates of seroma formation in the PDS mesh cohort may be explained, in part, by decreased inflammation around the prosthetic for a shorter period of time.

While rates of seroma were decreased in the PDS mesh cohort, there was only a trend of infection reduction that did not reach statistical significance. This may be because of the practice of early seroma aspiration by the surgical team, which could have prevented the evolution of seroma to infection. It is also possible that there was a reduction in clinical severity of infections reflected by the reduction in complication-associated implant removal with the use of PDS mesh. However, these differences in infection rate did not reach statistical significance. Future studies should aim to replicate this comparison of ADM with PDS mesh with larger cohorts to more thoroughly investigate the relationship between scaffolding material and infection.

The mechanism underlying the finding that the use of PDS mesh was independently associated with decreased risk of mastectomy flap necrosis compared to ADM was less clear. Numerous studies investigating ADM use in IBBR have noted the increased risk of mastectomy flap necrosis, which was consistent with our findings. 9 27 28 35 Mastectomy, which interrupts the axial perfusion of the breast skin, results in a relatively hypovascular skin flap that is supplied by the subdermal plexus and vulnerable to necrosis. 36 One hypothesis explaining increased rates of necrosis with ADM use implicates differences in intraoperative expansion and subsequent tension on the hypovascular mastectomy flap, leading to ischemia and eventual necrosis. 35 However, our study design using only a single surgeon to control for variations in surgical techniques, such as intraoperative expansion, makes this hypothesis less likely. Instead, we offer the hypothesis that differences in vascularization around the scaffolding structures underlie observed differences in mastectomy necrosis. Specifically, we posit that the FBR to ADM creates increased vascularity around the expander, which acts to divert perfusion to the overlying mastectomy flap. The influx of endothelial cells begins as early as 3 days after ADM implantation, followed by vascular luminal structures that begin to form by day 7. 37 PDS mesh, however, produces some vascularization with no broad bands of capillaries at 30 or 90 days after implantation. 16 It may be possible that the increased recruitment of endothelial cells and subsequent angiogenesis around the expander observed in ADM use, but not PDS mesh use, prevents adequate angiogenesis to perfuse the mastectomy flap, leaving the mastectomy flap prone to necrosis. The authors feel that it is important to emphasize that this is a preliminary hypothesis and that further studies are required to elucidate the true mechanism to explain differences in mastectomy flap necrosis.

Aside from the clinical differentiators of PDS mesh relative to ADM, there are some manufacturing and economic considerations. The PDS mesh—like other synthetic meshes—can be manufactured and sterilized with controlled and consistent parameters. ADM thickness may vary somewhat from its marketed specifications due to variations introduced during production stages, including tissue harvest, processing, and storage. 33 Synthetic mesh thickness is more reproducible and subject to the narrow constraints of the manufacturer's machining protocols. Secondly, the cost of PDS mesh will vary regionally and nationally in accordance to market conditions but is generally lower than the cost of ADM (at the senior author's institution), the ADM (FlexHD, Musculoskeletal Transplant Foundation, Edison NJ) cost ranges from $15.22 to $17.24 per square centimeter (cm), while the PDS mesh cost ranges from $6.00 to $8.00 per square cm.

There are a few studies that compare other synthetic meshes with ADM. Consistent with our findings, the use of polypropylene mesh has been associated with decreased rates of major complications, seroma, and prosthesis loss compared to ADM in primary and secondary subpectoral breast reconstructions. 38 One meta-analysis comparing ADM to absorbable or non-absorbable synthetic meshes demonstrates that rates of seroma were lower (OR: 0.2) with absorbable synthetic mesh. 39

There are key limitations to the present study. Firstly, all procedures included in the study were performed by a single surgeon at one institution who was a patent and equity holder in the synthetic PDS mesh and formerly a consultant and speaker for the ADM manufacturers. To try to limit this potential for bias in patient selection, we performed the analysis with propensity matching on consecutive patients. Propensity matching, which aims to create comparable groups between study cohorts based on demographic characteristics, is a validated tool in plastic surgery research. 40 41 42 43 44 45 46 47 To further limit the risk of bias in patient selection, the compared cohorts were both consecutive and exclusive. That is, patients from the transition period when both ADM and mesh were simultaneously used were excluded since bias could have existed in the choice of one over the other. Despite propensity matching, there were still differences in BMI, which limited our analysis. Second, it is possible that there were differences in mastectomy techniques used during this time period that confound differences in complication rates, such as seroma, wound dehiscence, and mastectomy flap necrosis. Specifically, we did not examine mastectomy skin incision type or mastectomy specimen weight, which would have helped deepen our understanding of differences between these two cohorts. Finally, we did not report any patient-reported outcomes, which also would have enhanced our analysis on the differences between PDS mesh and ADM for primary breast reconstruction.

With respect to the technique differences in the two cohorts, the PDS mesh cohort had more pre-pectoral reconstructions, in-line with the evolving mores of IBBR over this same time period. It is possible that selection bias towards those with thicker mastectomy flaps was present in determining those who received pre-pectoral reconstruction. However, the differences in pre-pectoral reconstructions could arguably *increase* complications in the mesh cohort since more mesh material is needed and the prosthesis is placed directly subjacent to hypovascular mastectomy flaps versus the submuscular approach, where the prosthesis has the more durable vascular coverage of muscle. It is also possible that the type of ADM could have impacted the complication difference since most of the ADM used was FlexHD, and there have been some reports that this has a higher complication rate than Alloderm (though other studies, including a randomized controlled trial, show these two ADMs have equivalent outcomes). 48 49 However, this potential difference in the ADM complication group was lower in magnitude than the stark difference in complications between the ADM and mesh groups as a whole. Secondly, the senior author's institution uses primarily non-fenestrated ADM, which may have a different safety and complication profile than fenestrated ADM. 50 Finally, all reconstructions with PDS mesh took place between 2021 and 2023, compared to reconstructions with ADM, which occurred as early as 2013. There may have been subtler differences in surgical practice, even within the single-surgeon control, that could confound these findings. A true randomized controlled trial would be superior to the current study of propensity-matched consecutive patients.

### Conclusion

Prosthetic breast reconstruction continues to evolve rapidly with new techniques and technologies. PDS is a new synthetic absorbable iteration of mesh-based implant reconstruction. Early experience shows that PDS mesh has an acceptable complication profile relative to ADM.
