# Supplementary material for: Early Experience with Synthetic Monofilament Polydioxanone Mesh in Primary Breast Reconstruction: A Propensity Matched Analysis
Source: Arch Plast Surg. 2026 Jun 17;53(4):321–30. doi: 10.1055/a-2850-0163 (PMC13496217; doi:10.1055/a-2850-0163)
Supplement: Supplementary file 1 [file 10-1055-a-2850-0163_29351464.pdf]

Supplementary Table 1. Demographic comparison of ADM and PDS mesh cohorts

		ADM (n=748)	PDS mesh (n=48)	p-value
Age		46.3 ± 10.6	43.0 ± 8.7	**0.04**
BMI		26.4 ± 6.0	23.7 ± 4.4	**< 0.001**
ASA status	I	54 (7.2%)	2 (4.1%)	0.09
	II	490 (65.5%)	39 (84.7%)	
	III	149 (19.9%)	7 (15.2%)	
Smoking	Never	546 (72.9%)	34 (73.9%)	0.74
	Current/former	202 (27.0%)	14 (30.4%)	
Diabetes		34 (4.5%)	0 (0%)	0.13
Mastectomy Reason	Prophylactic	344 (45.9%)	26 (54.1%)	0.27
	Therapeutic	404 (54.0%)	22 (47.8%)	
Radiation		130 (17.4%)	2 (4.2%)	**0.01**
Chemotherapy		301 (40.2%)	14 (30.4%)	0.13
Expander Plane	Prepectoral	138 (18.4%)	0 (0%)	**0.001**
	Subpectoral	610 (81.5%)	48 (100%)	
Mastectomy Type	SSM	440 (58.8%)	17 (44.7%)	**< 0.001**
	NSM	265 (35.4%)	31 (64.6%)	

ADM = acellular dermal matrix, ASA = American Society of Anesthesia, CI = confidence interval, NSM = nipple-sparing mastectomy, PDS = polydioxanone, ref. = reference, SSM = skin-sparing mastectomy

Supplementary Table 2. Results of Binary Logistic Regression to Predict Any Complication

		Odds Ratio	95% CI	p-value
Age		1.011	0.973 - 1.030	0.34
BMI		1.166	1.013-1.645	**0.02**
ASA Classification	I (ref.)			0.55
	II	1.123	0.542-2.542	0.59
	III	1.182	0.492-2.830	0.60
Diabetes		0.893	0.618-2.991	0.61
Current or Past Smoker vs Never Smoker (ref.)		2.319	1.892-3.247	**0.01**
Chemotherapy		0.982	0.482-1.702	0.69
Radiation		0.739	0.419-1.304	0.29
Therapeutic vs. Prophylactic Mastectomy (ref.)		1.362	0.896-1.947	0.83
PDS Mesh vs. ADM (ref.)		0.523	0.093-0.824	**0.01**
Prepectoral (ref.) vs. Subpectoral		0.281	0.114-0.827	**0.04**
SSM (ref.) vs. NSM		1.531	0.629-2.239	0.84

$\chi^2(15) = 95.95$, $p < 0.001$, Hosmer-Lemeshow statistic = 0.12, c-statistic = 0.77

ADM = acellular dermal matrix, ASA = American Society of Anesthesia, CI = confidence interval, NSM = nipple-sparing mastectomy, PDS = polydioxanone, ref. = reference, SSM = skin-sparing mastectomy

Supplementary Table 3. Results of Binary Logistic Regression to Predict Seroma

		Odds Ratio	95% CI	p-value
Age		1.014	0.994-1.033	0.77
BMI		1.241	0.856 - 1.457	0.18
ASA Classification	I (ref.)			0.91
	II	2.424	0.179 - 5.311	0.46
	III	2.084	0.402 – 7.238	0.32
Diabetes		0.841	0.211 - 1.232	0.41
Current or Past Smoker vs Never Smoker (ref.)		1.114	0.128 – 2.133	0.87
Chemotherapy		0.875	0.411 - 1.922	0.54
Radiation		0.622	0.214 – 1.003	0.06
Therapeutic vs. Prophylactic Mastectomy (ref.)		1.391	0.782 - 2.381	0.28
PDS Mesh vs. ADM (ref.)		0.414	0.182 - 0.781	**0.03**
Prepectoral (ref.) vs. Subpectoral		0.281	0.180 - 0.557	**0.01**
SSM (ref.) vs. NSM		0.821	0.424 - 1.972	0.36

$\chi^2(15) = 50.88$, $p < 0.001$, Hosmer-Lemeshow statistic = 0.874, c-statistic = 0.91

ADM = acellular dermal matrix, ASA = American Society of Anesthesia, CI = confidence interval, NSM = nipple-sparing mastectomy, PDS = polydioxanone, ref. = reference, SSM = skin-sparing mastectomy

Supplementary Table 4. Results of Binary Logistic Regression to Predict Expander Exposure

		Odds Ratio	95% CI	p-value
Age		1.146	0.838-1.857	0.65
BMI		1.433	1.094-1.883	**0.01**
ASA Classification	I (ref.)			0.40
	II	1.231	0.328-6.982	0.54
	III	1.782	0.346-5.783	0.51
Diabetes		0.591	0.187-3.524	0.68
Current or Past Smoker vs Never Smoker (ref.)		1.392	0.478-3.951	0.43
Chemotherapy		1.098	0.516-1.980	0.41
Radiation		0.520	0.173-1.895	0.36
Therapeutic vs. Prophylactic Mastectomy (ref.)		1.953	0.832-4.372	0.19
PDS Mesh vs. ADM (ref.)		0.256	0.193-0.959	**0.03**
Prepectoral (ref.) vs. Subpectoral		0.827	0.222-1.994	0.35
SSM (ref.) vs. NSM		1.008	0.233-3.012	0.40

$\chi^2(15) = 31.07$, $p = 0.009$, Hosmer-Lemeshow statistic = 0.99, c-statistic = 0.93

ADM = acellular dermal matrix, ASA = American Society of Anesthesia, CI = confidence interval, NSM = nipple-sparing mastectomy, PDS = polydioxanone, ref. = reference, SSM = skin-sparing mastectomy

Supplementary Table 5. Results of Binary Logistic Regression to Predict Complication-Associated Expander Removal

		Odds Ratio	95% CI	p-value
Age		1.421	0.233-1.923	0.84
BMI		1.246	1.134-1.952	**0.02**
ASA Classification	I (ref.)			0.14
	II	2.423	0.568-10.231	0.18
	III	3.899	0.957-12.234	0.27
Diabetes		0.942	0.199-2.923	0.65
Current or Past Smoker vs Never Smoker (ref.)		2.103	1.125-4.421	**0.02**
Chemotherapy		0.893	0.446-1.840	0.81
Radiation		1.109	0.891-1.348	0.33
Therapeutic vs. Prophylactic Mastectomy (ref.)		1.230	0.849-1.901	0.43
PDS Mesh vs. ADM (ref.)		0.201	0.032-0.792	**0.02**
Prepectoral (ref.) vs. Subpectoral		0.568	0.184-1.083	0.06
SSM (ref.) vs. NSM		1.241	0.823-2.932	0.45

$\chi^2(15) = 65.95$, $p < 0.001$, Hosmer-Lemeshow statistic = 0.56, c-statistic = 0.88

ADM = acellular dermal matrix, ASA = American Society of Anesthesia, CI = confidence interval, NSM = nipple-sparing mastectomy, PDS = polydioxanone, ref. = reference, SSM = skin-sparing mastectomy

Supplementary Table 6. Results of Binary Logistic Regression to Predict Mastectomy Flap Necrosis

		Odds Ratio	95% CI	p-value
Age		1.147	0.872 - 1.452	0.29
BMI		1.194	1.132 - 1.642	**0.01**
ASA Classification	I (ref.)			0.37
	II	1.392	0.453 - 4.277	0.52
	III	2.035	0.602 - 6.875	0.28
Diabetes		0.53	0.149 - 1.887	0.31
Current or Past Smoker vs Never Smoker (ref.)		2.827	1.759 - 4.781	**<0.001**
Chemotherapy		0.651	0.356 - 1.190	0.23
Radiation		0.940	0.406 - 2.176	0.83
Therapeutic vs. Prophylactic Mastectomy (ref.)		1.034	0.592 - 1.806	0.90
PDS Mesh vs. ADM (ref.)		0.109	0.014 - 0.901	**0.03**
Prepectoral (ref.) vs. Subpectoral		0.642	0.309 - 1.336	0.24
SSM (ref.) vs. NSM		2.237	1.181 - 4.014	**0.01**

$\chi^2(15) = 71.93$, $p < 0.001$, Hosmer-Lemeshow statistic = 0.613, c-statistic = 0.89

ADM = acellular dermal matrix, ASA = American Society of Anesthesia, CI = confidence interval, NSM = nipple-sparing mastectomy, PDS = polydioxanone, ref. = reference, SSM = skin-sparing mastectomy

Supplemental Table 7. Results of Binary Logistic Regression to Predict Infection

		Odds Ratio	95% CI	p-value
Age		1.391	0.931 - 1.294	0.13
BMI		1.194	1.074 - 1.267	**0.01**
ASA Classification	I (ref.)			0.25
	II	1.528	0.182 - 12.828	0.70
	III	0.582	0.054 - 6.218	0.65
Diabetes		1.393	0.279 - 6.950	0.69
Current or Past Smoker vs Never Smoker (ref.)		2.394	0.899 - 4.902	0.13
Chemotherapy		1.262	0.513 - 3.106	0.61
Radiation		0.534	0.130 - 2.191	0.38
Therapeutic vs. Prophylactic Mastectomy (ref.)		0.968	0.393 - 2.387	0.94
PDS Mesh vs. ADM (ref.)		0.582	0.072 - 4.711	0.61
Prepectoral (ref.) vs. Subpectoral		0.318	0.092 - 0.832	**0.03**
SSM (ref.) vs. NSM		1.351	0.473 - 3.858	0.57

$\chi^2(15)$ =31.13, p = 0.001, Hosmer-Lemeshow statistic = 0.19, c-statistic = 0.97

ADM = acellular dermal matrix, ASA = American Society of Anesthesia, CI = confidence interval, NSM = nipple-sparing mastectomy, PDS = polydioxanone, ref. = reference, SSM = skin-sparing mastectomy
